# Indents

**Published:** 1921-05

**Authors:** 


### INDENTS

**☞Brief Articles of Technical or Scientific Value will receive notice and comment. Contributions invited.**

---

SPARE THE TOOTH AND SOIL THE FAD. Are we to become a toothless nation? That sounds extreme, but toothless we shall become unless a saving sense intervenes and curbs the tendency of physicians and dentists to ascribe most of our ills to germs that lie in dental ambush. It would seem that the path of this latest scientific innovation has been blazed by the X-ray. Formely a germ was permitted to thrive unseen. Unhonored by Roentgen photography, a tooth remained. Today the dentist who has glimpsed the base of a tooth on an X-ray plate is like the impressionable young man who has seen the picture of a beautiful girl—he is not satisfied until he sees the original. So out comes the tooth. That is, if the patient is lucky. For usually it is teeth.

This is not the heresy of a layman. Dr. H. V. Fuqua, president of the Chicago Dental Society, calls the present extracting tendency a fad and believes that it will pass with other fads. But when the fad passes, the teeth sacrificed in the dental rites will not return. They are ''spurlos versenkt,'' with a reverse English.

In connection with the subject at hand, it might be well to quote from a letter received at the office of the *Dental Facts* from one of its professional readers in the Southwest, who speaks out of the fullness of his professional career, covering a period of thirty years:

''The fad of focal infection has bred an excess of exodontists who are working their lack of conscience overtime to prey on the unsuspecting whom they have wrought up to a frenzy on the question of the systemic effect of focal infection. The claim that arthritis, rheumatism, endocarditis and other complaints (from biliousness and stomachache) are largely caused by infected teeth is impressed on the credulous by citing a few cases where removal of teeth has been followed by restored health; as if that had been all that had been needed for the regaining of health in those particular cases. The extremists never mention other causes of ill health that were removed at the same time, nor do they speak of the innumerable cases where the patient, on their advice, had many teeth extracted without any apparent improvement.

Every ethical dentist knows of these cases. The exodontist plays on the ignorance of the people to enable him to extract their teeth, and at the same time 'pull their leg,' which he does by shutting his eyes and making a charge. I have known exodontists to extract healthy teeth, where there was absolutely no reason for doing so, unless it was that they might 'possibly some time' cause trouble.

The exodontist enjoys blocking nerves just to show how much can be done without pain. And at 'five per' he will extract anything that suits his fancy. This is easily proven by the examination of the teeth 'before and after' they are out. How far does the 'five per' impel him? In many cases to and even beyond the legal limit.

I have sent patients to extraction specialists and have regretted it, for they did not get the conscientious service they had a right to expect.

Now, what can an ethical dentist do to curb the questionable practice of the profiteering exodontists? I, for one, will not refer a patient to them without definite instructions. I also inform the patient at what point the exodontist becomes liable for damages and subject to recovery by law.

Many diseased teeth need extracting, but all pulpless teeth do not. Any man who practices good dentistry can exhibit patients with pulpless teeth who have had them ten, twenty or more years, who, in spite of these pulpless teeth, are as healthy as their neighbors with all live natural or with all store teeth.

The weight of the diagnosis is on the lay dentist and not on the exodontist at 'five per,' and when he is no longer permitted to extract the 'five to fifty per,' he will become more trustworthy.

What thinks the lay dentist?

Prague, Okla. (Signed) DR. G. E. ZINN."

It is hoped that the readers of this will understand that nothing whatever can be said against radiography as radiography and its usefulness to the world. It is, no doubt, one of the great discoveries of the age, and much has already been and much more is still to be accomplished with it. Likewise, the skilled exodontist who serves conscientiously and honestly those who are referred to him, is a godsend to any community. His services, when backed by years of earnest preparation and study, together with months of apprenticeship, under a preceptor of wide experience, can not be criticised. The point is, however, that it is a pity that so great a thing, so eminent a service, should have been prostituted and made to pander to the greed of some few individuals. As our good friend Dr. Zinn writes complaining of.—F. W. Mowbray, in *Dental Facts.*

---

CAST FULL UPPER DENTURE WITH SPECIAL ATTENTION GIVEN TO ANATOMICAL FORM AND PHYSIOLOGICAL FUNCTION OF THE SOFT TISSUES. When we start to criticize the work of nature, developed in normal form with the view to increase function by making improvements in these forms, we had better stop before we begin. And it amounts to the same thing as criticism when we unthinkingly substitute other forms for hers. The only anatomical form of which I wish to speak and which is to be reproduced in dentures, either cast or vulcanite, is the form of the soft tissue of the mouth. An analysis of what we do when restoring contour of the face is nothing more than the restoration of natural labial contours within the mouth. This contour of the labial surface is more within the realm of the aesthetic than that of function.

Funtionally, a denture is placed for speech and mastication. From the point of view of speech the palatal surface should reproduce the form of the vault in contour so that the proper size and shape of air spaces can easily be formed by the tongue. The rugae acts as high points to enable the tongue to do this more accurately. The convex roll just distal to the anterior teeth is essential for use in making the escapement and explosive sounds, and as this roll continues distally along the bicuspids and molars, it makes the correct contact with the sides of the tongue easy. However, I consider that the most important form for functioning is the abrupt contour of tissue adjacent to the gingival margin, which acts as an auxiliary masticating surface, a shortstop to the food to prevent it from being forced too far palatally. We find that in normal teeth the forms are such that the excursions of food through the embrasures and along the palatal grooves are such as to massage this most important tissue and keep it in a healthy tone. If this tissue is of sufficient importance for nature to make a tooth form for its care and health, surely it is of importance enough for us to reproduce. Not only does the construction of this form act as a shortstop, but we are enabled to make more normal palatal tooth surfaces and to make the teeth stand out and function more normally. (Every little thing even though of small consequence in itself should be incorporated in the finished denture; for at best we know that the efficiency of an artificial denture falls far short of ideal function.) Some one has compared the rugae to a plasterer's mortar board—the plasterer, in handling his mortar, throws it against the board, but he places it differently on his trowel. In a similar manner the tongue places the food against the rugae in order for it to take a new hold and present it for chewing from a new angle. Let nature's form be your guide in forming denture contours.—Dr. C. J. Stansbery, in *Journal N. D. A.*

---

Do Not Use Lubricants on Siliceous Fillings. When a matrix is used during the insertion of a siliceous cement filling, never put any lubricant on it. The smallest amount passing into the filling will seriously injure it. A clean, dry celluloid strip can be used to bring the filling to place and make the contour, and if it is held firmly for two or three minutes it can be removed without injuring the filling.—Robt. J. Cruise, in *Dental Facts.*

---

Contact Points on Silicate Fillings. In filling proximal cavities with silicate materials, sufficient separation should be obtained to allow for thickness of celluloid strip. If not, no matter how perfect the margins, that filling is a failure, because the all-important contact point is lost. Contact point (hand-made) should be, nearly as is possible, like the geometrical "point." In some silicate fillings that point extends from incisal to gingival margin.—C. M. C., in *Dental Facts.*

---

Cementing an Inlay. In cementing a gold inlay, malleting the inlay by using a stiff, strong orange-wood stick will give excellent results in seating inlay. This method is followed by rigid burnishing and finally having patient put full strength of muscles in closing teeth after a piece of orange-wood about one-eighth inch long and squared on all sides has been placed on the inlay. This throws full pressure on the inlay, tending to drive inlay further to place and overcomes any possibility of heaving while cement is crystalizing.—W. D. N. Moore, in *Dental Facts.*

---

To Restore Lower Incisors. Worn lower incisors in the mouths of elderly patients present a trying problem. Often they are abraded to a point of extreme sensitiveness, yet the pulps are not exposed, and devitalization and subsequent pulp removal is very difficult. Gold crowns are possible in some cases, but are always dirty and unsightly. Yet many of these cases may be handled by trimming the tooth on both mesial and distal surfaces with a thin carborundum stone, thus making the tooth bluntly wedge-shaped.

Lightly groove these ground surfaces with a fissure bur, taking care to make groove as nearly as possible parallel with the long axis of the tooth. (These light grooves serve in the finished inlay to prevent displacement in either anterior or posterior direction, and also act as guides when the inlay is placed in position.)

These preparatory steps having been taken, prepare and cast an inlay in the usual manner. Great care should be taken with the finishing when the work has been set to see that no slightly rough margin offers a chance for careless use of floss or pick to flip the inlay from its slender anchorage. Advantages of this method are that it conserves the vital pulp, requires little drilling, and in a portion of the tooth where it is most easily tolerated, is very serviceable, and presents a minimum of gold.—Arthur E. Smith, Peoria, Ill., in *Dental Review.*

---

GUM TISSUE GRAFTING. I wonder if other dentists would be interested in grafting gum tissue. I had a patient, Mrs. F., who had been wearing a porcelain crown for some years on the right superior central, and in biting a piece of peanut brittle she broke the root and process clear to the apex, leaving a very bad case for bridgework or plate. She had a very short upper lip so I was in a quandary as to what to do. I used novocain in the surrounding tissue, took a sharp lance and scraped off all the outer surface of the gum. Then I cut several pieces of gum from the six and twelve-year molars, pressed into place on freshened gum where the central had been broken out until the contour of the gums was perfect, took cotton roll saturated with oil of eucalyptus and pressed gums into place, then flowed collodion five or six times to hold loose pieces of the gums in place. Well, to cut a long story short, I had and have had perfect success in all cases, not a single bit of suppuration and the gums healed perfectly, and do not show scarred tissue. I have not seen a discussion of mouth grafting in any of the journals.—Dr. F. E. DeLane, *Dental Digest.*

---

To Stop Leakage in a Slightly Worn Hypodermic. Many good glass-barreled syringes used for conduction and local anesthesia are discarded after a period of usage because they develop a slight leakage when the plunger is set in motion (the syringe being filled with the injecting solution), the leakage occurring between the plunger and the barrel due possibly to wear. This leakage can be overcome by having the plunger goldplated by a jeweler at a cost of a dollar or probably less. This idea was suggested to me some years ago by Dr. Geo. A. Coleman, Philadelphia. —Dr. J. J. MacMillan, *Cosmos.*

---

To Etch the Surface of a Gold Inlay. Moisten the surface not protected with wax with seventy-five per cent. hydrochloric acid and then treat with mercury. After the surface has the desired etching, heat the inlay in an open flame to drive off the mercury and treat with the usual solutions. This gives a finely crystalline surface to which the cement will adhere.—C. J. Hadley, *Pacific Dental Gazette.*

---

Narrow Bicuspid Spaces in Bridgework. How often we find the bicuspids must be replaced with a bridge from first molar to cuspid and the space is too narrow for two bicuspids and too wide for one. Such cases can be artistically restored by using a thick, wide molar facing which fills the space, grinding the buccal and occlusal surfaces, to resemble two bicuspids. Use a sharp knife-edged stone while grinding buccal surface. It is not necessary to cut through the facing; simply grind to give it the appearance of two bicuspids, then finishing the lingual and occlusal surfaces in the usual manner by casting. By this method one platinum pin is near the center of each bicuspid reproduction. If two narrow bicuspid facings are used they look like rat teeth. Grind a wide molar facing by this method and keep for future reference.—W. O. Fellman, *Dental Facts.*

---

PHYSICAL EXERCISES AND THE "MIDDLE-AGED MAN." At the College of Ambulance, Queen Anne Street, Sir James Cantlie has opened what is lightheartedly described in the hopeful press as "a new crusade against old age." In giving his blessing to this scheme, which appears to comprise a series of exercises suitable for middle-aged persons of sedentary habits, Sir James maintained that the man of forty or fifty should be at his best. As a rule he is not, because he feels, when he becomes middle-aged, that he must give up the activities of his youth. He makes the fatal mistake, it seems, of sinking into an easy-chair and taking little or no exercise, with the result that he usually develops rheumatism and dies years before there is the slightest necessity to die. The preventives of old age prescribed by Sir James Cantlie are so simple that it seems almost incredible that any ordinary man should die before he is eighty at the least.

This is what he has to say to the middle-aged: "Don't think you ought to be dignified; play about with the children. Avoid easy-chairs and carpet slippers. Take plenty of exercise of the right kind. Never think you are too old at seventy. Don't give rheumatism—the chief English complaint—a chance to develop. Take down the old warming pan from the wall, and use it to dry the bed.

Dry your pajamas and underclothing every day before you put them on.'' We might add to Sir James Cantlie's modest list another item, and that would be: When you are forty or fifty, don't imagine you are twenty or thirty. In the brittle period of middle age it is almost as easy to bring about disaster by attempting too much as by attempting too little. Not a few middle-aged men who served in the army, and were absolutely fit to do their own military job, began, after many years of abstinence, to play Association and even Rugby football, and in many cases they were entirely incapacitated in consequence. It is a very good thing to urge men of all ages to take methodical exercise, diet themselves rationally, and to take other elementary precautions to keep themselves in good health. But there is rather an ill-considered tendency at the present time to suggest that there exists some magic formula by which a man need never be so old as he is. Physical fitness is a relative condition, having a somewhat different significance at different ages. We know of no arbitrary device, including the rapturously advertised thyroid gland, which will give back youth to old age. ''Putting back the clock'' is a pleasant fiction; and the experiences of men famous in the ring or in other branches of sport and athletics who have sought to ''come back'' have not been encouraging. Middle age is too often old age, and youth is sometimes middle age; but middle age can not be youth.—*Hospital.*

---

Tobacco Smoke as a Mouth Disinfectant. Professor V. Puntoni, of the University of Rome, has undertaken some experiments with the object of ascertaining the real action of tobacco smoke as a disinfectant under conditions similar to those which exist in the oral cavity. The results of these experiments are interesting. In the first place, it was found that the strikingly disinfectant power that tobacco smoke exercised in vitro did not occur to the same extent in the mouth of the smoker, and the most that could be said was that a bactericidal action was only shown to follow the consumption of very large quantities of tobacco, and then only on the micro-organisms of least resistance, such as the meningococcus and cholera vibrio. It is not admissible that microbes having the resistance of *B. typhosus* or greater can be killed in the mouth by tobacco smoke, and it is absurd to think that the bactericidal action of the smoke could manifest itself in the respiratory tract as a sequel to inhalation. The different qualities of tobacco made use of in these experiments showed a disinfecting power almost equal in relation to the weight of tobacco used; denicotinised cigars acted just as powerfully as ordinary ones. The smoke of tobacco completely decolorised by filtration through compressed cotton-wool retained a marked bactericidal action notwithstanding the loss of all the nicotine and tar products, which are the two elements possessing definite disinfecting power. The bactericidal substances contained in this decolorised smoke are soluble in water, one of them being capable of distillation at 100 degrees C. and identical with formaldehyde, the other not capable of distillation was pyrrol, the bactericidal action of which as a component of tobacco smoke, and hitherto unknown, is important.—*Dental Record.*

